# Morphological Changes of Anterior Cerebral Artery (ACA) in Hydrocephalic Pediatric Patients

**Published:** 2017

**Authors:** Sait OZTURK, Erdogan AYAN, Metin KAPLAN

**Affiliations:** 1 Firat University, School of Medicine, Department of Neurosurgery, Elazig, Turkey; 2 Namık Kemal University, School of Medicine, Department of Neurosurgery, Tekirdag, Turkey

**Keywords:** Anterior cerebral artery, Cerebral perfusion, Evans’ Index, Hydrocephalus

## Abstract

**Objective:**

The morphology of anterior cerebral artery (ACA) in patients with hydrocephalus (HCP) was analyzed, and its importance was discussed in maintaining cerebral perfusion.

**Materials & Methods:**

A total of 84 cases in 2 groups between 0 and 3 months, followed-up at Firat Universitesi Hastanesi, Beyin Cerrahisi Klinigi, Elazig, Turkiye due to in 2010-2013, were enrolled. Two groups were created for the study. Group 1; patients with HCP and Group 2; as control group without HCP. In both groups, the length of the A2 segment of ACA was measured from its origin to the junction of the genu and body portions of the corpus callosum on T2 mid-sagittal magnetic resonance (MR) scans. For all cases, axial MR imaging scans were used to calculate Evans’ index (EI), and the cases were divided into three groups: Group A, EI ≥50%; Group B, EI of 40-50% and Group C, EI <40%. The two groups (Groups 1 and 2) were compared with respect to ACA length, and the correlation with the EI was quantified. P values below 0.05 were considered statistically significant.

**Results:**

Mean length of ACA was 57.3 mm in Group 1 and 37.5 mm in Group 2. EI increased as the length of ACA increased. A statistical comparison of the two groups revealed that the ACA length was significantly greater in Group 1. The relationship between EI and ACA length was statistically significant.

**Conclusion:**

Reducing ventricular size appears to be an important factor in addition to reducing intracranial pressure in an attempt to maintain normal cerebral perfusion(CP).

## Introduction

Vascular insufficiency is associated with embryological dysgenesis, acquired stenosis due to atherosclerosis, and increased intracranial pressure. In particular, space-occupying lesions are associated with compression and shifting to adjacent vessels. Another important condition that requires attention is the transposition of great vessels caused due to shifts in brain structures (1). The compression, shift, and transposition of the vascular structure interfere with blood flow dynamics and negatively affect cerebral perfusion (CP) (2). Poor outcomes are inevitable if such pathological processes occur in major vascular branches. Increased intracranial pressure further complicates vascular insufficiency. 

Many studies have measured cerebral blood flow (CBF) using transcranial doppler (TCD) ultrasonography, and the results of these studies have made significant contributions to the explanations of many pathological processes (3, 4). Blood flow in major cerebral vessels has been measured using TCD in patients with hydrocephalus (HCP) (1, 5, 6). The use of TCD in newborns and infants is more efficient due to structural advantages of the neonate cranium (7, 8). Ventricle enlargement shifts the anatomic positions of intracranial structures. Although many studies have evaluated blood flow using TCD in patients with HCP, there are no reports regarding anterior cerebral artery (ACA) morphology. 

In the present study, we evaluated ACA morphology in patients with HCP using cranial magnetic resonance (MR) images.

## Materials & Methods

The subjects were divided into two groups for the purpose of the study. 

**Group 1 (Patients with hydrocephalus):** A total of 84 cases between 0 and 3 months, ollowed-up at Firat Universitesi Hastanesi, Beyin Cerrahisi Klinigi, Elazig, Turkey, due to HCP between 2010 and 2013, were evaluated for their eligibility to participate in the present study. Among these patients, only 22 cases, in which the ACA was clearly visualized on T2-weighted mid-sagittal MR scans, were enrolled (Table 1); the remaining 62 patients were excluded. Other than the MRI scans originally performed for the current medical condition, no additional investigations to visualize ACA, such as MR angiography or computerized tomography angiography were requested, due to ethical concerns. Only the existing cranial MR scans were evaluated. Of these cases, 15 had myelomeningocele, 4 had hemorrhagic disease of the newborn, and 3 cases had isolated HCP caused by intrauterine factors. 

The study was approved by the local Ethics Committee and informed consent was taken from subjects parents.

**Table 1:** Gender, Age, Diagnosis at Admission, Evans Index and Length of Anterior Cerebral Artery of the Patients

**Patients**	**Gender**	**Age (days)**	**Diagnosis**	**EI (%)**	**LACA (mm)**
**Group 1** **1**	**M**	**87**	**MMC**	**69.9**	**76.2**
**2**	F	3	MMC	57.2	79.0
**3**	F	62	HCP	54.0	67.0
**4**	F	2	MMC	51.8	71.0
**5**	F	5	MMC	51.7	94.4
**6**	M	6	HCP	51.0	46.8
**7**	M	1	MMC	50.9	68.0
**8**	M	4	MMC	49.2	62.7
**9**	F	11	HDN	48.9	59.3
**10**	M	4	MMC	48.8	43.2
**11**	F	4	MMC	48.3	51.0
**12**	M	3	MMC	47.7	54.0
**13**	M	9	MMC	47.2	57.3
**14**	M	1	MMC	46.3	73.0
**15**	F	4	MMC	45.9	42.0
**16**	F	11	HDN	45.1	39.8
**17**	F	1	HDN	45.0	40.5
**18**	F	3	HDN	42.9	52.6
**19**	M	14	MMC	42.8	63.0
**20**	M	1	HCP	42.4	39.8
**21**	F	4	MMC	42.0	42.6
**22**	M	6	MMC	41.6	39.0
**Group 2** **1**	**F**	**20**	**Epilepsy**	**29.4**	**40.0**
**2**	M	16	ICSOL	17.3	42.0
**3**	M	90	Epilepsy	27.9	38.0
**4**	F	14	Epilepsy	23.9	34.0
**5**	M	27	ICSOL	25.0	36.0
**6**	F	8	MMR	25.9	41.0
**7**	M	42	Epilepsy	22.8	37.0
**8**	M	4	MMR	25.0	36.0
**9**	F	27	ICSOL	24.4	35.1
**10**	M	63	ICSOL	25.2	36.2

**EI: **Evans index, **F:** Female,** HCP:** Hydrocephalus, **HDN:** Hemorrhagic disease of the newborn, **ICSOL:** Suspicion of intracranial space occupying lesion, **LACA: **Length of anterior cerebral artery, **M: **Male, **MMC:** Myelomeningocele, **MMR:** Suspicion of mental-motor retardation, **%:** Percent, **mm:** Millimeter

**Table 2:** The Mean Length of Anterior Cerebral Artery (ACA) in Sub-Divided Evans Index (EI) Groups

**EI values**	**The length of ACA**
EI > 50% (Gr A)	71.7 mm*
EI between 50%-40% (Gr B)	50.6 mm*
EI < 40% (Gr C)	37.5 mm*

(*) **p<0.005**

**Fig 1.**
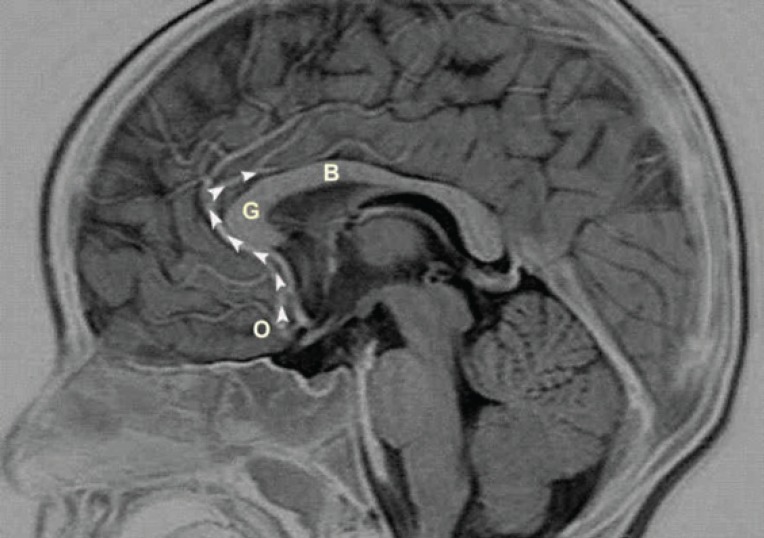
Mid-sagittal T2-weighted negative picture of cranial magnetic resonance image. The length of anterior cerebral artery has been measured from origo of anterior cerebral artery to junction of genu and body of corpus callosum (white arrows). **B: **Body of corpus callosum, **G: **Genu of corpus callosum, **O: **Origo of anterior cerebral artery

**Fig 2:**
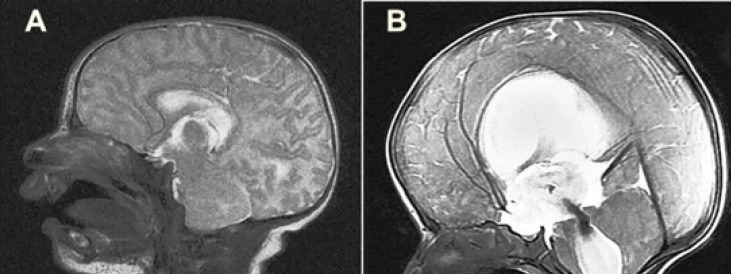
Mid-sagittal T2-weighted cranial magnetic resonance images of a patient with the diagnosis of myelomeningocele. **(A) **At newborn period. **(B) **The image of same patient which taken two months after birth showed that hydrocephalus, increased length of anterior cerebral artery and apparent tension of the vascular structures

**Group 2 (Control group):** A total of 10 pediatric patients between 0 and 3 months, who had undergone cranial MR imaging for reasons other than HCP and in which ACA was clearly visualized in T2-weighted midsagittal sequences, were randomly selected from the radiology archives as the control group (Table 1). 

**Measurement of the ACA length: **The length of the ACA was measured from its origin to the junction of the genu and body portions of the corpus callosum (A2 segment of ACA) (Figure 1) (9). In all cases, the length of the ACA was measured and recorded using a measurement program at the workstation (Advantage Windows, software version 4.2, GE Healthcare, Little Chalfont, UK) used to evaluate MRI scans from a 1.5-Tesla MR imaging device (Signa Excite, GE Healthcare).

**Evans’ index and relation with the ACA length: **For all cases (Group 1 and 2), axial MR imaging scans were used to calculate Evans’ index (EI), and the cases were divided into three groups: Group A, EI ≥50%; Group B EI of 40-50%; Group C EI<40%. The two groups (Groups 1 and 2) were compared with respect to ACA length, and the correlation with the EI was quantified.

**Statistical Analysis:** A standard SPSS 12.0 for Windows (SPSS Institute, Chicago, IL, USA) software package was used for all statistical analyses. The ACA length was compared between the groups using a MannWhitney U test. In the analysis of the study population sub grouped according to the EI, Kruskal-Wallis analysis of variance was initially used to examine the relationship between EI and the ACA length. Then, a Mann-Whitney U test with Bonferroni correction was used because a statistically significant difference was observed between the groups. P values below 0.05 were considered statistically significant. 

## Results

The mean ACA length was 57.3 mm (max: 76.2 mm, min: 39.0 mm) in patients with HCP (Group 1) and 37.5 mm (max: 42.0 mm, min: 32.0 mm) in the control group (Group 2). A statistical comparison of the two groups revealed that the ACA length was significantly greater in patients with HCP (P<0.05) (Table 1) (Figure 2). The mean ACA length was 71.7 mm in patients with EI ≥50% (Group A), 50.6 mm in patients with EI from 40- 50% (Group B), and 37.5 mm in the 10 patients in the control group who did not have HCP (Group C) (Table 2). Notably, EI increased along with the ACA length. The increase in ACA length was particularly striking in patients with EI ≥50%. The relationship between EI and ACA length was statistically significant (P<0.05). 

## Discussion

Hydrocephalus causes a shifting of brain structures due to increased ventricular volume and higher intracranial pressure. Both conditions result in demyelization, particularly in the periventricular area, and cellular death in the long term, which finally leads to irreversible damage. CP plays an important role in the HCP-induced damage (10, 11). Increased intracranial pressure reduces CP, so restoring CP by decreasing intracranial pressure is a critical step in the treatment of HCP. Changes occurring in CP help determine whether cerebrospinal fluid (CSF) drainage would be beneficial in patients with ventriculomegaly (5, 11). For example, higher rates of shunt complications have been reported in patients with ventriculomegaly and preserved CBF (10, 11). For this reason, changes in ACA blood flow have been assessed using CP TCD in infants with HCP (12). In particular, decreased end diastolic flow velocity of CBF and increases in pulsatility and the resistive index have been reported in patients with HCP (5, 11, 13). CSF drainage is followed by decreased CBF velocity and decreases in pulsatility and the resistive index (13, 14). Increased intracranial pressure plays an important role in establishing vascular resistance (RI) against arterial blood flow. The vasculature can be considered as a system with laminar flow, and numerous factors in a laminar flow system can alter flow dynamics (15). These factors include fluid viscosity, the roughness of the inner surface of the lumen, angulations, length, and the diameter of the lumen (Poiseuille’s law) (15). Marked elongation, stretching, and configuration changes were observed in the ACAs of patients with HCP (Figure 2). In particular, decreased lumen diameter caused by vessel elongation and stretching would result in an increase in RI. ACA blood flow is more complicated than a simple laminar flow system due to the presence of vascular tonus and the effects of sympathetic and parasympathetic stimuli. In our opinion, the hemodynamic response to stimuli is altered in abnormally elongated and stretched vascular structures. 

Our results reveal that important morphological changes occur in patients with HCP. Changes in ACA morphology became more prominent as the EI increased. We hypothesize that morphological changes of the ACA in patients with HCP play an important role in decreasing CP. Furthermore, the present study also offers a new perspective regarding the relationship between HCP and CP. The evaluation and reporting of ACA morphology in the routine radiologic examination of patients with HCP would improve patient treatment and follow-up.

Limitation of this report is CBF could not be measured with TCD in concordance with MRI scans for ethical reasons. Sedation of the patients required for optimal blood flow measurement with TCD. In addition, there are a great many articles in the literature regarding CBF of ACA in hydrocephalic patients. The main aim of this study was evaluation morphological changes of ACA behind the CBF of ACA. 

In patients with HCP, changes in ACA morphology became more prominent as the EI increased. We simply state that the A2 is longer in patients with hydrocephalus.

**In conclusion,** reducing ventricular size appears to be an important factor in addition to reducing intracranial pressure in an attempt to maintain normal CP. The reversal of morphological changes caused by elongating, stretching, and shifting the vascular structures would be beneficial for restoring normal CP. 
